# Targeted RNA Therapy Reprograms Fibrotic Macrophages to Reverse Pulmonary Fibrosis

**DOI:** 10.7150/thno.132296

**Published:** 2026-06-04

**Authors:** Zhimin Song, Jingjing Chen, Jiaying Fan, Tinghong Zhang, Xing Peng, Yao Pan, Yun Zhang, Yaofeng Wang, Jinling Qin, Shu Meng

**Affiliations:** 1 State Key Laboratory of Respiratory Disease, the First Affiliated Hospital, Guangzhou Medical University, Guangzhou, Guangdong, 510120, China.; 2 Department of Basic Science Research, Guangzhou National Laboratory, Guangzhou, Guangdong, 510005, China.

**Keywords:** pulmonary fibrosis, macrophage reprogramming, immunometabolism, RNA therapeutics, arginase-1

## Abstract

**Rationale:**

Pulmonary fibrosis is driven by maladaptive immune programs that reinforce fibroblast activation. We sought to determine whether profibrotic macrophage states could be therapeutically remodeled to alleviate fibrosis through targeted RNA-based modulation of their intrinsic regulatory circuitry.

**Methods:**

Integrated single-cell transcriptomic and metabolomic analyses were performed to identify regulatory programs associated with profibrotic macrophage states during lung fibrosis. A mannose-functionalized lipid nanoparticle platform was developed to preferentially deliver regulatory RNAs to CD206⁺ macrophages *in vivo*. Therapeutic efficacy was evaluated by Arg1 silencing or targeted delivery of *Irf5* mRNA to assess macrophage state remodeling in fibrotic lungs.

**Results:**

Transcriptomic and metabolomic analyses identified an Arg1-centered metabolic module that stabilizes profibrotic macrophage states during lung fibrosis. The mannose-functionalized lipid nanoparticle system enabled preferential RNA delivery to CD206⁺ macrophages *in vivo*. Arg1 silencing disrupted profibrotic metabolic programs, whereas targeted delivery of *Irf5* mRNA transcriptionally redirected macrophages toward an inflammatory, antifibrotic state, resulting in attenuation of pulmonary fibrosis.

**Conclusions:**

These findings demonstrate that macrophage states can be therapeutically remodeled through targeted RNA-based modulation in pulmonary fibrosis. This strategy establishes state-level immune reprogramming as a potentially generalizable approach for dismantling pathogenic immune programs in fibrotic disease.

## Introduction

Pulmonary fibrosis is a progressive lung disease characterized by chronic tissue injury, aberrant repair, and excessive extracellular matrix (ECM) deposition 1-4. Macrophages are central regulators of fibrotic progression 5, 6, and experimental depletion of macrophages alleviates fibrotic pathology across multiple organs 7, 8. However, resident macrophages are indispensable for tissue homeostasis and host defense. Thus, a major unresolved challenge is how to selectively target profibrotic macrophage programs without compromising essential macrophage functions.

Macrophage functional states are often broadly dichotomized into classically activated (M1) and alternatively activated (M2) phenotypes; however, macrophages *in vivo* are highly heterogeneous and exist along a continuum. M1 macrophages exhibit inflammatory and microbicidal functions 6. In contrast, M2 macrophages are strongly associated with fibrotic disease, serving as a major source of profibrotic cytokines and promoting ECM deposition and remodeling 6, 9. Arginase-1 (Arg1), a canonical marker of M2 macrophages, is a key metabolic enzyme governing arginine utilization 10, 11. By converting arginine to ornithine, Arg1 supports proline synthesis and collagen production, thereby directly linking macrophage metabolism to fibrotic remodeling 12. These observations suggest that disrupting M2-associated metabolic programs, or enforcing a shift toward an M1-like state, may represent a rational therapeutic strategy for fibrosis.

Macrophage state transitions are orchestrated by transcription factors (TFs). Multiple TF families, including signal transducers and activators of transcription (STATs), nuclear factor kappa B (NF-κB), activator protein 1 (AP-1), interferon regulatory factors (IRFs), hypoxia-inducible factors (HIFs), peroxisome proliferator-activated receptors (PPARs), glucocorticoid receptors (GRs), and Krüppel-like factors (KLFs), have been implicated in M1 polarization 13, among which IRF5 plays a dominant role in promoting M1 identity 14, 15. RNA therapeutics have emerged as a powerful modality for cell-state transitions 16, 17. Notably, mannose-based delivery systems enable preferential targeting of CD206⁺ macrophages, an M2-enriched population, within fibrotic tissues 18, thereby providing a strategy for cell-state-selective intervention.

Here, we investigate whether pathogenic macrophage states in pulmonary fibrosis can be therapeutically dismantled and redirected by targeting distinct layers of their regulatory architecture. Using integrated single-cell transcriptomic and metabolomic analyses, we identify Arg1 as selectively enriched in CD206⁺ M2 macrophages during fibrotic progression and examine the impact of Arg1 silencing on macrophage state stability and fibrotic remodeling. In parallel, we test whether targeted delivery of *Irf5* mRNA to CD206⁺ macrophages can transcriptionally reprogram macrophage identity and modulate fibrotic progression. By applying this platform to deliver either siRNA or mRNA, this study explores RNA-based strategies to selectively perturb fibrotic macrophage states and provides a framework for state-level immune reprogramming in fibrotic disease.

## Results

### Arginine metabolism represents a dominant metabolic alteration in lung fibrosis

**Figure 1:**
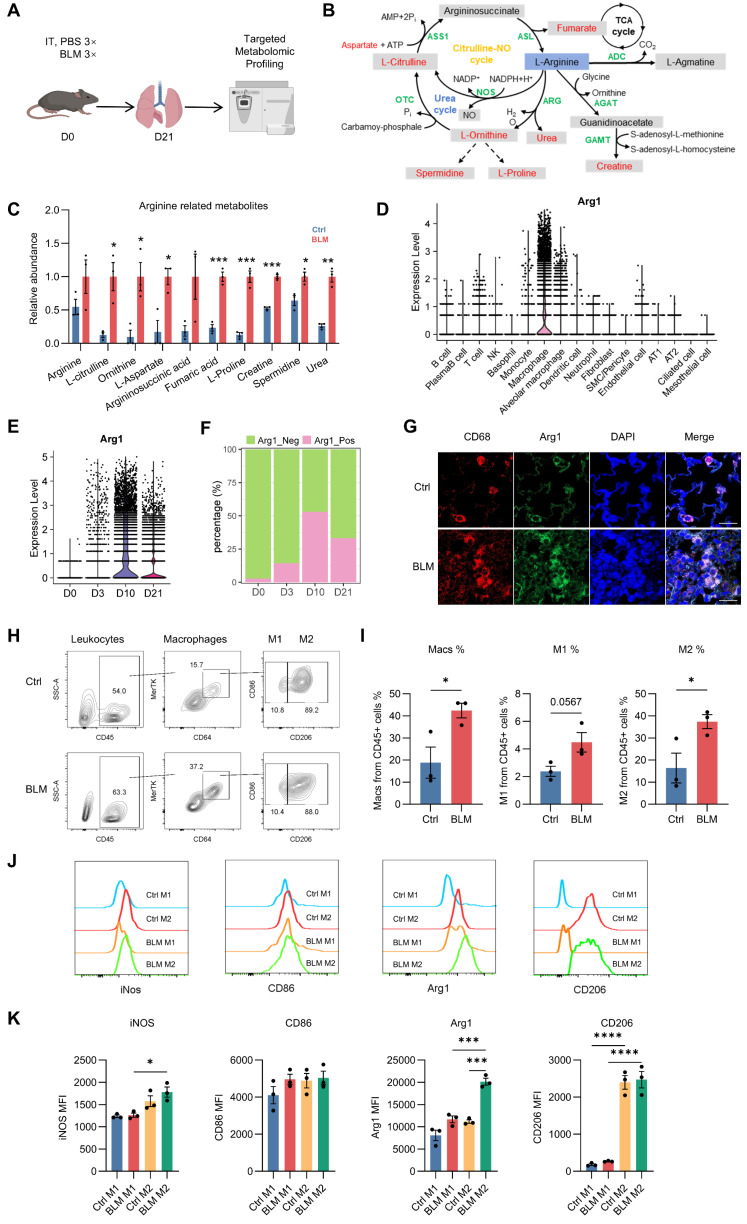
** Integrated metabolomic and single-cell analyses reveal Arg1-centered arginine metabolism in fibrotic lung macrophages.** A, Schematic illustration of the experimental design. Mice were treated with PBS or bleomycin, and lung tissues were collected at day 21 for targeted metabolic profiling. B, Diagram depicting key enzymatic steps and metabolites involved in arginine metabolism, highlighting pathways relevant to arginine utilization and downstream metabolic flux. C, Bar plot showing the relative abundance of arginine-related metabolites. n = 3; *P < 0.05, **P < 0.01, ***P < 0.001. D, Violin plots showing *Arg1* expression across major lung cell types based on single-cell RNA sequencing analysis. E, Violin plots showing temporal changes in *Arg1* expression in lung macrophages at days 0, 3, 10, and 21 following bleomycin administration. F, Bar plot quantifying the proportions of *Arg1*⁺ and *Arg1*⁻ macrophages as a percentage of total lung macrophages. G, Representative immunofluorescence images showing Arg1 expression in lung macrophages (CD68⁺) from PBS- or BLM-treated mice at day 10; scale bar, 20 μm. H, Representative flow cytometry plots identifying M1 (CD45⁺CD64⁺MerTK⁺CD206⁻) and M2 (CD45⁺CD64⁺MerTK⁺CD206⁺) macrophages. I, Quantification of total macrophages as a percentage of CD45⁺ cells and the relative proportions of M1-like and M2-like macrophages among CD45⁺ cells (n = 3); Student’s t test, *P < 0.05. J, Representative flow cytometry histograms showing expression of iNOS, CD86, Arg1, and CD206 in M1-like and M2-like macrophages from PBS- or BLM-treated mice at day 10. K, Quantification of mean fluorescence intensity (MFI) of iNOS, CD86, Arg1, and CD206 in M1-like and M2-like macrophages under PBS and BLM conditions at day 10 (n = 3); one-way ANOVA, *P < 0.05, ***P < 0.001, ****P < 0.0001.

To define metabolic alterations associated with lung fibrosis, we performed targeted metabolomic profiling of lung tissues from control and bleomycin (BLM)–treated mice at day 21 after injury (Figure 1A). Global metabolite analysis revealed broad metabolic remodeling in fibrotic lungs, with most detected metabolites showing increased abundance in BLM-treated samples, as visualized by volcano plots and heatmaps (Figure S1A-B). Notably, amino acids and amino acid–derived metabolites represented a prominent subset of the upregulated metabolites (Figure S1B-C).

Pathway enrichment analysis further identified arginine metabolism as a major axis of metabolic dysregulation in fibrotic lungs. Analyses using both the Small Molecule Pathway Database (SMPDB) and KEGG consistently highlighted arginine- and nitrogen-related pathways among the most altered, including the urea cycle, arginine biosynthesis, and arginine/proline metabolism (Figure S2A-B). Interestingly, these pathways are directly linked to Arg1 catalytic activity and the downstream generation of ornithine and proline, metabolites that support collagen synthesis and extracellular matrix remodeling. Consistent with these pathway-level changes, quantitative analysis demonstrated altered abundance of multiple arginine-derived metabolites in BLM-treated lungs (Figure 1B-C).

Together, these data place Arg1-associated arginine metabolism within a broader metabolic reprogramming landscape during lung fibrosis, providing a metabolic context for subsequent analysis of Arg1 regulation and function in fibrotic macrophages.

### Dynamic enrichment of Arg1 in lung M2 macrophages during fibrotic progression

To define the cellular sources and temporal dynamics of Arg1 expression during lung fibrosis, we performed and analyzed single-cell RNA sequencing data from mouse lungs collected at 0, 3, 10 and 21 days following BLM injury. Arg1 expression was predominantly confined to macrophages and was only sparsely detected in non-macrophage lung cell populations (Figure 1D), consistent with its established role as a marker of alternatively activated (M2) macrophages. Arg1 levels in lung macrophages peaked at day 10 after BLM administration and remained elevated during the fibrotic phase (Figure 1E–F). Immunofluorescence staining showed elevated Arg1 expression in CD68⁺ lung macrophages from BLM-treated mice at day 10 (Figure 1G).

To delineate macrophage subsets, we assessed the expression of C-type lectin receptor CD206 19, a canonical M2 marker, and CD86, a canonical M1 marker. Whereas CD86 was broadly expressed across lung macrophages and failed to clearly segregate subsets, CD206 expression robustly separated macrophages into two distinct populations (Figures S3 and 1H). We therefore defined CD45⁺CD64⁺MerTK^+^CD206⁺ macrophages as M2 and CD45⁺CD64⁺ MerTK^+^CD206⁻ macrophages as M1 for subsequent analyses.

Flow cytometric analysis revealed an increased proportion of macrophages among CD45⁺ leukocytes following BLM injury, accompanied by a marked expansion of the M2 macrophage population at day 10 after treatment (Figure 1I). Notably, Arg1 expression was markedly enriched in M2 macrophages in BLM-treated lungs compared with controls (Figure 1J-K).

Together, these data demonstrate that Arg1 is dynamically and selectively enriched in CD45⁺CD64⁺ MerTK^+^CD206⁺ M2 lung macrophages during fibrotic progression, supporting a close association between Arg1 induction and profibrotic macrophage states.

### Development of LNP-Mannose for M2 macrophage-targeted RNA delivery

**Figure 2:**
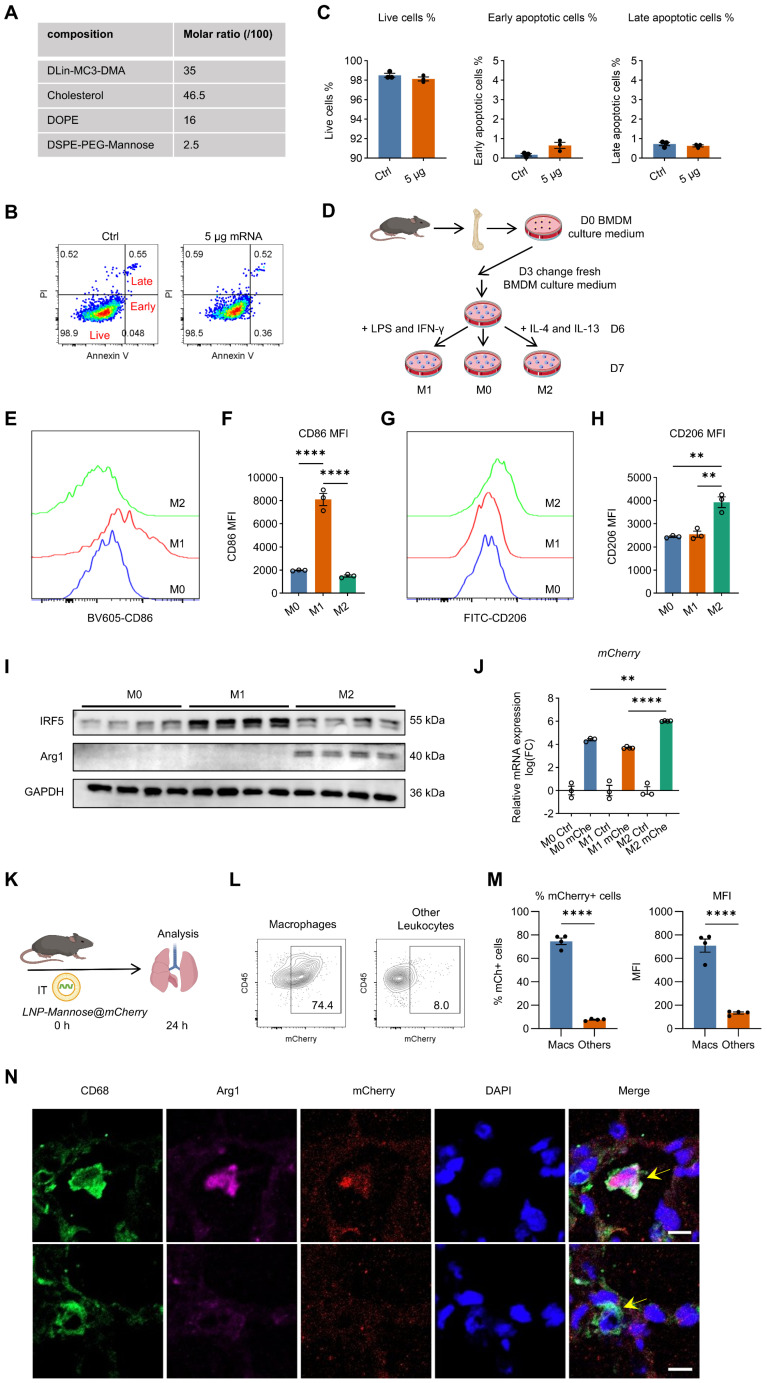
** LNP-Mannose preferentially targets mRNA delivery to M2 macrophages.** A, Composition of LNP-Mannose. B, Representative flow cytometry plots of apoptosis in RAW264.7 cells transfected with or without 5 µg mCherry mRNA. C, Quantification of live, early apoptotic, and late apoptotic cells assessed by flow cytometry (n = 3; mean ± s.e.m.; one-way ANOVA; *P < 0.05, **P < 0.01). D, Schematic of *in vitro* differentiation of mouse bone marrow–derived macrophages (BMDMs) into M1 or M2 macrophages. E, Representative flow cytometry plots showing CD86 expression in M0, M1, and M2 macrophages. F, Mean fluorescence intensity (MFI) of CD86 in M0, M1, and M2 macrophages (n = 3; mean ± s.e.m.; one-way ANOVA; ****P < 0.0001). G, Representative flow cytometry plots showing CD206 expression in M0, M1, and M2 macrophages. H, MFI of CD206 expression in M0, M1, and M2 macrophages (n = 3; mean ± s.e.m.; one-way ANOVA; **P < 0.01). I, Representative immunoblot showing IRF5 and Arg1 expression in BMDMs following stimulation with LPS/IFN-γ or IL-4/IL-13. J, qPCR analysis showing increased mCherry expression in M2 compared with M0 or M1 macrophages 24 h after LNP-Mannose–mediated mCherry mRNA transfection (n = 3; mean ± s.e.m.). K, Schematic of intratracheal delivery of LNP-Mannose–encapsulated mCherry mRNA *in vivo*. L, Representative flow cytometry plots showing mCherry expression in macrophages and other leukocytes. M, Quantification of the percentage of mCherry^+^ cells and mean fluorescence intensity in macrophages, PMNs, and other leukocytes (n ≥ 3; mean ± s.e.m.; one-way ANOVA; **P < 0.01, ****P < 0.0001). N, Representative immunofluorescence images showing selective mCherry expression in M2 macrophages (CD68⁺Arg1⁺), but not in other macrophage subsets (CD68⁺Arg1^-^). Scale bar, 20 μm.

To enable targeted RNA delivery to M2 macrophages and potentially disrupt M2 function or promote an M2-to-M1 phenotypic switch, we developed a macrophage-targeted lipid nanoparticle platform. Previous studies have shown that the mannose receptor CD206, a canonical M2 marker, mediates mannose recognition and uptake 20. Based on this principle, we engineered mannose-modified lipid nanoparticles (LNP-Mannose) composed of DLin-MC3-DMA, cholesterol, DOPE 21, and DSPE-PEG-mannose (Figure 2A). The polydispersity indices (PDIs) of both LNP-Mannose and LNP-Mannose@mCherry were below 0.2, indicating a narrow size distribution. The Z-average diameter increased from 147.1 nm for LNP-Mannose to 184.7 nm after mCherry loading, while the zeta potential shifted from 14.04 mV to -4.76 mV (Figure S4A–E).

We first assessed the cytotoxicity of LNP-Mannose *in vitro*. Delivery of up to 5 μg mCherry mRNA into 1 × 10⁵ RAW264.7 cells did not induce apoptosis and resulted in minimal cytotoxicity, as determined by Annexin V and propidium iodide staining (Figure 2B–C), indicating that LNP-Mannose is well tolerated.

To assess *in vivo* safety, LNP-Mannose was administered intratracheally, and major organs were collected for histological analysis 24 h later. HE staining of the heart, liver, spleen, lung, and kidney revealed no detectable histopathological differences between LNP-Mannose and PBS-treated groups (Figure S5), supporting the safety of this formulation.

To evaluate delivery efficiency and cell-type specificity, bone marrow–derived macrophages (BMDMs) were polarized into M1 or M2 phenotypes using LPS plus IFN-γ or IL-4 plus IL-13, respectively (Figure 2D). Successful polarization was confirmed by the robust induction of IRF5 and CD86 in M1 macrophages, and Arg1 and CD206 in M2 macrophages (Figures 2E–I and S6). Consistent with CD206-mediated uptake, LNP-Mannose–mediated delivery of mCherry mRNA resulted in significantly higher mCherry expression in M2 macrophages compared with unpolarized M0 macrophages or M1 macrophages, demonstrating preferential uptake by M2 macrophages *in vitro* (Figure 2J).

We next examined the *in vivo* targeting specificity of LNP-Mannose following intratracheal administration of mCherry mRNA. Flow cytometric analysis revealed that approximately 75% of lung macrophages were mCherry⁺ at 24 h after delivery, whereas only ~8% of other leukocyte populations exhibited detectable mCherry expression. Mean fluorescence intensity analysis further confirmed substantially higher mCherry signal in macrophages compared with other immune cell types (Figures 2K-M and S7). Immunofluorescence analysis of lung sections corroborated these findings, showing selective mCherry expression in CD68⁺Arg1⁺ M2 macrophages, with minimal signal detected in CD68⁺Arg1⁻ macrophages or non-macrophage cells (Figure 2N). Consistent with the macrophage-selective delivery, little to no mCherry signal was observed in PECAM1⁺ endothelial cells, EpCAM⁺ epithelial cells, α-SMA⁺ cells, or PDGFR-β⁺ fibroblasts (Figure S8).

Together, these data demonstrate that LNP-Mannose enables efficient and targeted mRNA delivery to lung macrophages *in vivo*, with a strong preference toward CD206⁺ M2 macrophages, establishing this platform as an effective strategy for macrophage-targeted RNA delivery in fibrotic lungs.

### M2 macrophage–targeted Arg1 siRNA therapy destabilizes M2 cell identity and attenuates lung fibrosis

**Figure 3:**
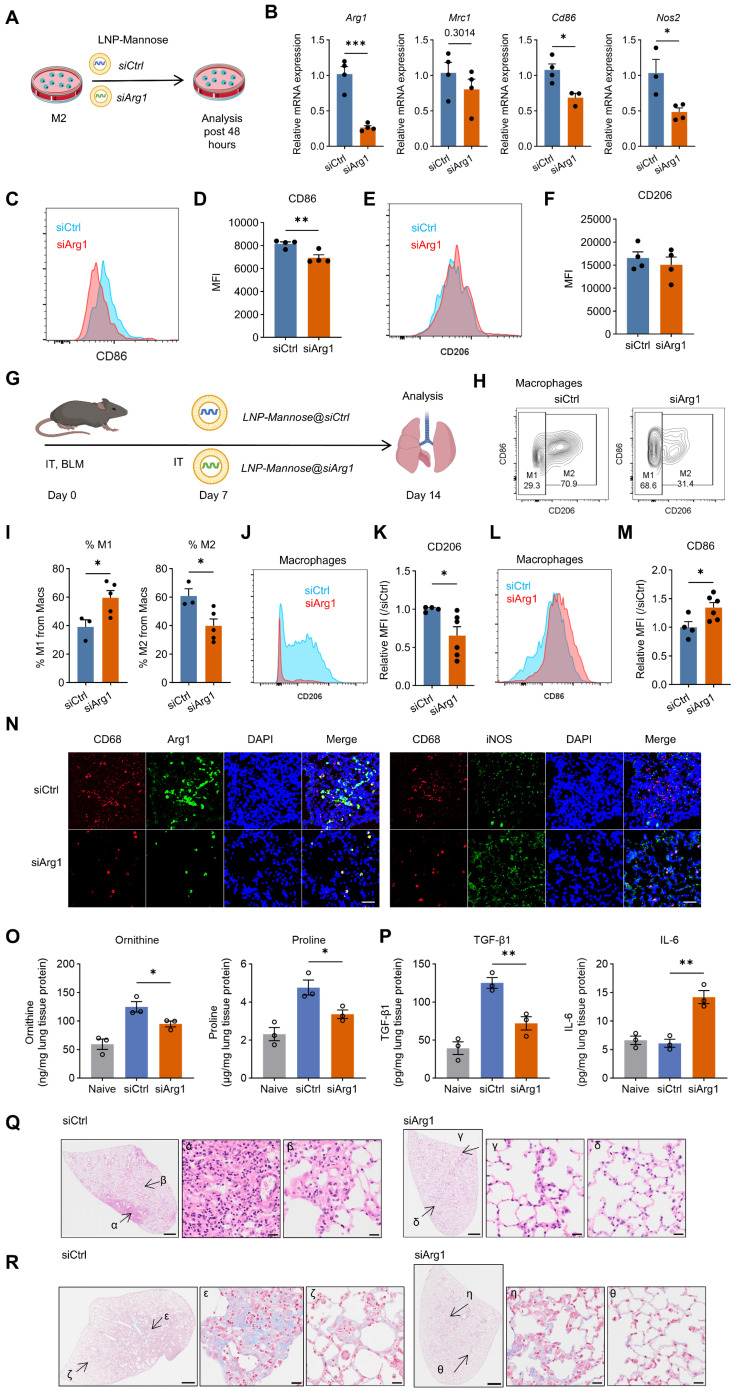
**LNP-Mannose–mediated Arg1 silencing drives macrophage phenotypic reprogramming and mitigates bleomycin-induced pulmonary fibrosis.** A, Schematic illustrating LNP-Mannose–mediated delivery of siCtrl or siArg1 into M2 macrophages; cells were analyzed 48 h after transfection. B, qPCR analysis of representative M1 and M2 marker gene expression following siCtrl or siArg1 transfection (n ≥ 3; mean ± s.e.m.; Student’s t test; *P < 0.05, ***P < 0.001). C, Representative flow cytometry plots showing reduced expression of the M1 marker CD86 after siArg1 transfection. D, Quantification of CD86 mean fluorescence intensity (MFI) (n = 4; mean ± s.e.m.; Student’s t test; **P < 0.01). E, Representative flow cytometry plots showing that CD206 expression is not affected by siArg1 transfection. F, Quantification of CD206 MFI (n = 4; mean ± s.e.m.; Student’s t test). G, Experimental scheme showing i.t. instillation of BLM (2.5 U/kg), followed by intratracheal administration of LNP-Mannose–encapsulated siCtrl or siArg1 on day 7; mice were analyzed on day 14 after BLM treatment. H, Representative flow cytometry plots showing a reduced proportion of lung M2 macrophages after LNP-Mannose–mediated siArg1 delivery compared with siCtrl. I, Quantification of the percentage of M1 and M2 macrophages in the lung by flow cytometry (n ≥ 3; mean ± s.e.m.; Student’s t test; *P < 0.05). J, Representative flow cytometry plots showing reduced CD206 expression in total lung macrophages in the siArg1 group compared with the siCtrl group. K, Quantification of CD206 MFI in total lung macrophages (n ≥ 4; mean ± s.e.m.; Student’s t test; *P < 0.05). L, Representative flow cytometry plots showing increased CD86 expression in total lung macrophages in the siArg1 group compared with the siCtrl group. M, Quantification of CD86 MFI in total lung macrophages (n ≥ 4; mean ± s.e.m.; Student’s t test; *P < 0.05). N, Representative immunofluorescence images showing reduced Arg1 expression and increased iNOS expression in lung macrophages following siArg1 delivery compared with siCtrl. O-P, Quantification of ornithine, proline, TGF-β1, and IL-6 levels in lung tissue homogenates (n = 3, mean ± s.e.m., Student’s t test; *P < 0.05, **P < 0.01). Q, Representative HE–stained lung sections showing reduced cellular accumulation in the interstitial area after siArg1 treatment. R, Representative images showing decreased collagen deposition in the lung interstitium following siArg1 delivery. Q-R, Scale bars, 1000 μm (whole tissue) and 20 μm (enlarged areas).

To selectively suppress Arg1 expression, we delivered siArg1 into M2-polarized BMDMs using the LNP-Mannose platform (Figure 3A). At 48 h after transfection, *Arg1* mRNA levels were significantly reduced in the siArg1 group compared with siCtrl. Concurrently, expression of the M2 marker *Mrc1* remained largely unchanged, whereas the M1-associated genes *Cd86* and *Nos2* were modestly reduced (Figure 3B). Flow cytometric analysis revealed decreased CD86 surface expression, while CD206 expression was maintained (Figure 3C–F), indicating that Arg1 silencing destabilizes the M2 transcriptional program rather than actively inducing M1 polarization *in vitro*.

We next assessed the therapeutic impact of Arg1 silencing *in vivo*. LNP-Mannose–encapsulated siArg1 was administered intratracheally at day 7 after BLM-induced lung injury, and lungs were analyzed at day 14 (Figure 3G). siArg1 treatment significantly reduced the proportion of M2 macrophages in fibrotic lungs (Figure 3H-I) and was associated with decreased CD206 and increased CD86 expression in lung macrophages (Figure 3J–M). Notably, the *in vivo* increase in CD86 expression contrasted with its reduction observed *in vitro*, suggesting that the effect of Arg1 silencing on CD86 is context-dependent and influenced by the fibrotic lung microenvironment. Immunofluorescence analysis further confirmed reduced Arg1 expression and increased iNOS expression following siArg1 delivery (Figure 3N).

To determine whether Arg1 silencing altered arginine metabolism and the lung microenvironment, we measured arginine and its downstream metabolites, together with selected fibrosis- and inflammation-associated cytokines, in lung homogenates. Compared with siCtrl, siArg1 significantly reduced ornithine and proline levels, whereas arginine levels remained unchanged, indicating decreased conversion of arginine into downstream profibrotic metabolites (Figures 3O and S9). This metabolic shift is consistent with the Arg1 knockdown phenotype. In parallel, siArg1 decreased TGF-β1 levels and increased IL-6 levels in lung tissue homogenates, while PDGF-BB, TNF-α, IL-10, and IL-1β levels were not significantly altered (Figures 3P and S9).

Histopathological analysis further demonstrated that M2-targeted Arg1 silencing markedly alleviated fibrotic pathology. siArg1-treated lungs exhibited reduced interstitial cellular accumulation, as assessed by HE staining (Figure 3Q), and attenuated collagen deposition, as shown by Masson’s trichrome staining (Figure 3R).

Together, these results demonstrate that selective Arg1 silencing in CD206⁺ M2 macrophages destabilizes the M2 program *in vivo*, shifts macrophage phenotypic balance, and significantly attenuates lung fibrosis.

### *Irf5* mRNA therapy reprograms M2 macrophages toward an M1 state and ameliorates lung fibrosis

**Figure 4:**
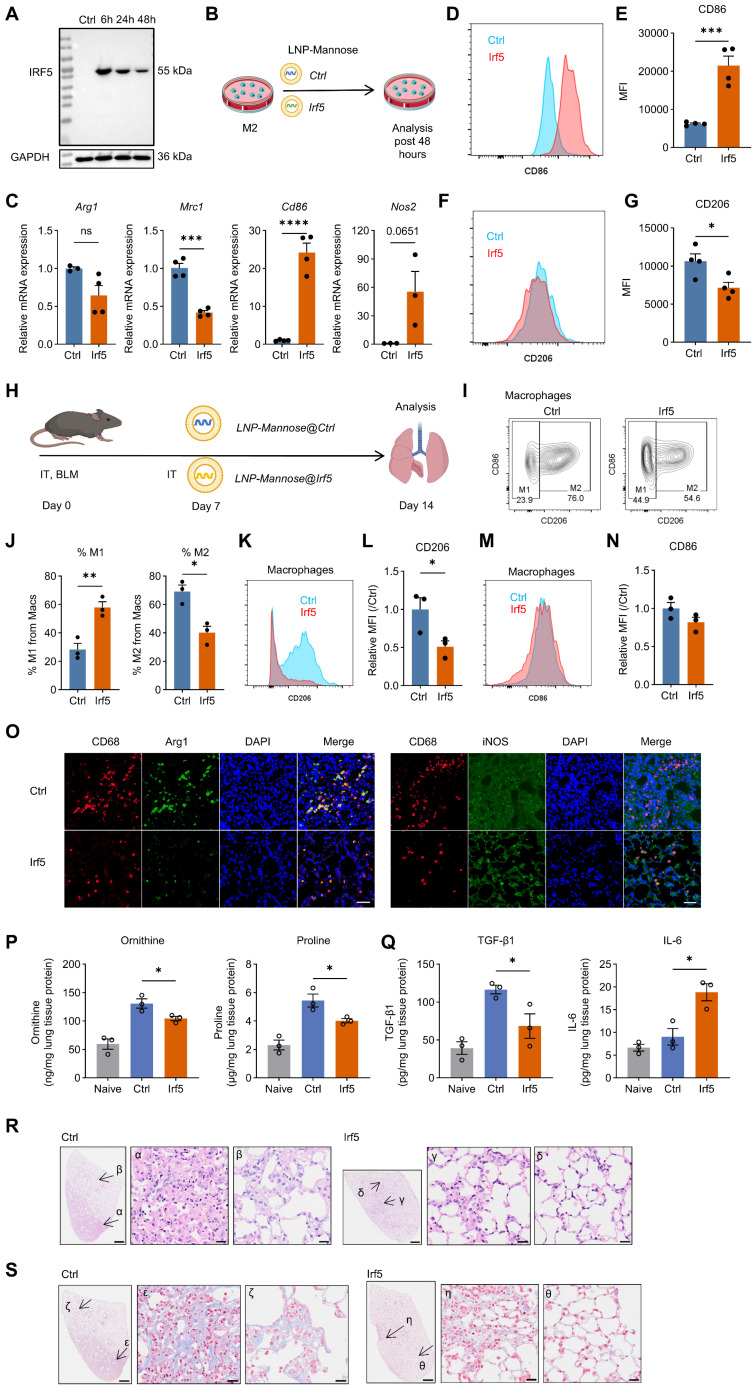
** LNP-Mannose–mediated *Irf5* mRNA delivery promotes macrophage repolarization and ameliorates bleomycin-induced lung fibrosis.** A, Representative immunoblot showing IRF5 expression in HEK293T cells following IVT *Irf5* mRNA transfection. B, Schematic illustrating LNP-Mannose–mediated delivery of vehicle control or *Irf5* mRNA into M2 macrophages; cells were analyzed 48 h after transfection. C, qPCR analysis of representative M1 and M2 marker gene expression following *Irf5* mRNA transfection (n ≥ 3; mean ± s.e.m.; Student’s t test; ***P < 0.001, ****P < 0.0001). D, Representative flow cytometry plots showing increased CD86 expression after *Irf5* mRNA transfection. E, Quantification of CD86 MFI (n = 4; mean ± s.e.m.; Student’s t test; ***P < 0.001). F, Representative flow cytometry plots showing reduced CD206 expression after *Irf5* mRNA transfection. G, Quantification of CD206 MFI (n = 4; mean ± s.e.m.; Student’s t test; *P < 0.05). H, Experimental scheme showing i.t. instillation of BLM (2.5 U/kg), followed by intratracheal administration of LNP-Mannose–encapsulated vehicle or *Irf5* mRNA on day 7; mice were analyzed on day 14 after BLM treatment. I, Representative flow cytometry plots showing the proportion of lung M1 and M2 macrophages following LNP-Mannose–mediated *Irf5* mRNA delivery compared with vehicle. J, Quantification of the percentage of M1 and M2 macrophages in the lung by flow cytometry (n = 3; mean ± s.e.m.; Student’s t test; *P < 0.05, **P < 0.01). K, Representative flow cytometry plots showing CD206 expression in total lung macrophages following *Irf5* mRNA delivery compared with vehicle. L, Quantification of CD206 MFI in total lung macrophages (n = 3; mean ± s.e.m.; Student’s t test; *P < 0.05). M, Representative flow cytometry plots showing comparable CD86 expression in total lung macrophages between *Irf5* mRNA–treated and vehicle groups. N, Quantification of CD86 MFI in total lung macrophages confirming no significant change following *Irf5* mRNA delivery (n =3; mean ± s.e.m.; Student’s t test). O, Representative immunofluorescence images showing reduced Arg1 expression and increased iNOS expression in lung macrophages after *Irf5* mRNA delivery compared with vehicle. P-Q, Quantification of ornithine, proline, TGF-β1, and IL-6 levels in lung tissue homogenates (n = 3, mean ± s.e.m., Student’s t test; *P < 0.05). R, Representative HE–stained lung sections showing modestly reduced cellular accumulation in the interstitial area following *Irf5* mRNA treatment. S, Representative images showing reduced collagen deposition in the lung interstitium after *Irf5* mRNA delivery. R-S, Scale bars, 1000 μm (whole tissue) and 20 μm (enlarged areas).

After targeting Arg1 to disrupt the profibrotic metabolic program, we asked whether imposing a defined transcriptional program could actively redirect macrophage identity to alleviate lung fibrosis. To this end, we ectopically expressed IRF5, a transcription factor that promotes M1 macrophage differentiation 14. *Irf5* mRNA was synthesized by *in vitro* transcription and its expression was validated in HEK293T cells (Figure 4A). LNP-Mannose–mediated delivery of vehicle or *Irf5* mRNA into M2-polarized macrophages resulted in a significant reduction of *Mrc1* expression together with increased *Cd86* mRNA levels (Figure 4B-C). These transcriptional changes were corroborated at the protein level by flow cytometry, which showed decreased CD206 and increased CD86 expression (Figure 4D-G).

We next assessed whether enforced *Irf5* expression could reprogram macrophages and mitigate fibrotic progression *in vivo*. LNP-Mannose–encapsulated *Irf5* mRNA was administered intratracheally at day 7 after bleomycin injury, and lungs were analyzed at day 14 (Figure 4H). *Irf5* mRNA delivery significantly reduced the abundance of M2 macrophages in fibrotic lungs compared with vehicle-treated controls (Figure 4I-J), indicating disruption of the M2-dominant macrophage landscape during fibrosis.

Phenotypic analysis revealed reduced CD206 expression in total lung macrophages following *Irf5* mRNA treatment, whereas CD86 surface expression remained largely unchanged (Figure 4K-N). Notably, immunofluorescence analysis demonstrated increased iNOS expression accompanied by reduced Arg1 expression in lung macrophages (Figure 4O), consistent with functional reprogramming toward an M1-like inflammatory state rather than complete surface marker conversion.

To examine how *Irf5* overexpression reshaped the metabolic and cytokine milieu in fibrotic lungs, we quantified arginine metabolites and inflammatory and fibrotic cytokines in lung homogenates following LNP-Mannose-mediated *Irf5* mRNA delivery. *Irf5* mRNA significantly reduced ornithine and proline abundance without altering arginine levels, indicating suppression of Arg1-associated downstream metabolic output. This was accompanied by decreased TGF-β1 and increased IL-6 and TNF-α levels, whereas PDGF-BB, IL-10, and IL-1β did not change (Figures 4P-Q and S9).

Importantly, this macrophage reprogramming was associated with attenuation of fibrotic pathology. Histological analyses revealed reduced interstitial cellular accumulation and decreased collagen deposition in *Irf5* mRNA–treated lungs, as assessed by HE and Masson’s trichrome staining, respectively (Figure 4R-S).

Together, these results demonstrate that macrophage-targeted *Irf5* mRNA delivery reprograms M2 macrophages toward an M1-like functional phenotype and alleviates lung fibrosis *in vivo*.

### Sustained antifibrotic effects of siArg1 and *Irf5* mRNA delivery in late-stage BLM-induced lung fibrosis

**Figure 5:**
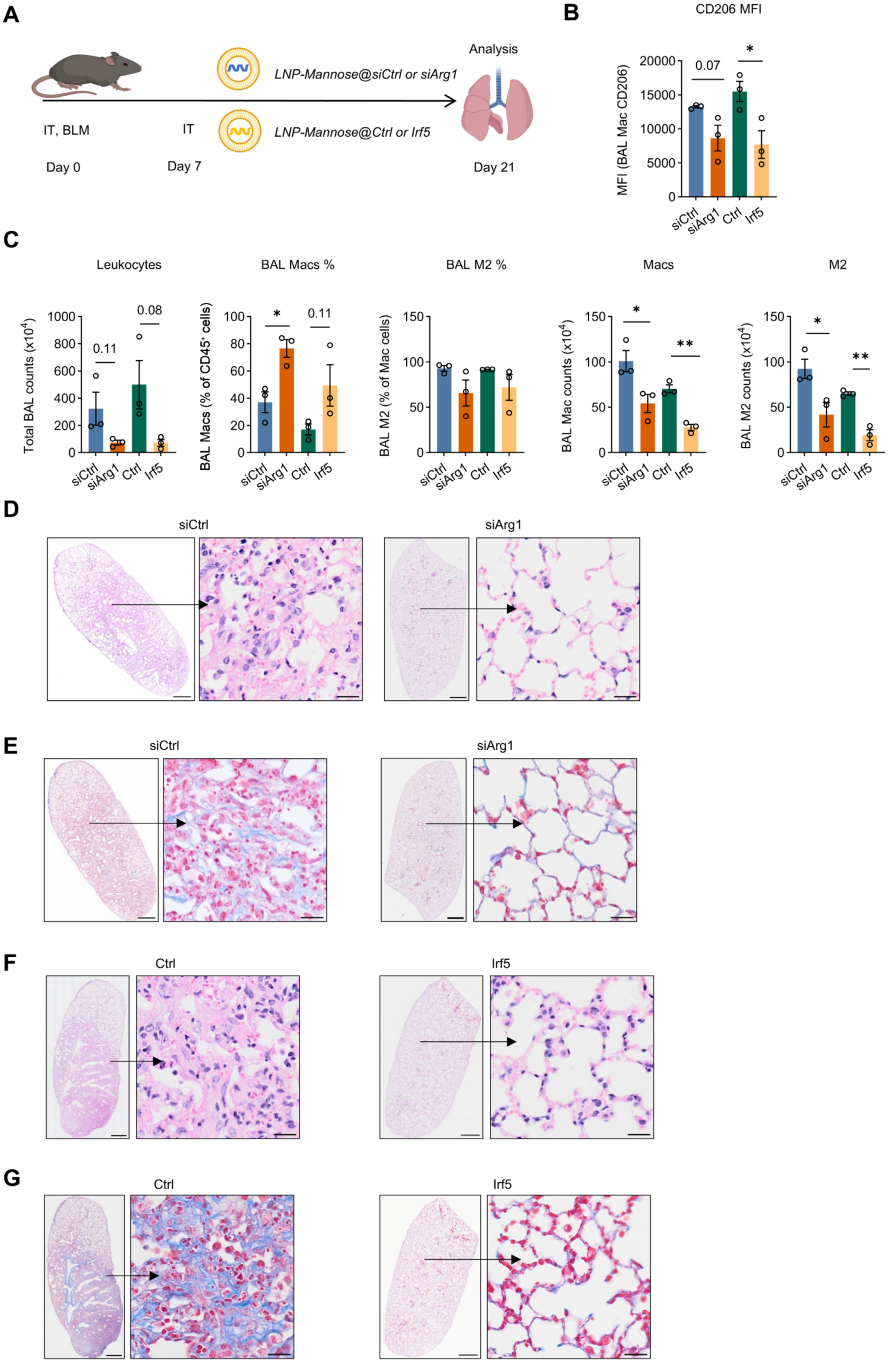
** Sustained antifibrotic effects of siArg1 and *Irf5* mRNA delivery during the late phase of BLM-induced lung fibrosis.** A, Schematic of the experimental design for intratracheal delivery of LNP-Mannose–encapsulated siArg1 or *Irf5* mRNA on day 7 after bleomycin (BLM) challenge, with analysis performed on day 21. B, Quantification of CD206 expression in BAL macrophages from the indicated groups at day 21 (n = 3; mean ± s.e.m.; Student’s t test; *P < 0.05). C, Quantification of total leukocyte number, percentage of macrophages, percentage of M2 macrophages among total macrophages, macrophage number, and M2 macrophage number in BAL from the indicated groups at day 21 (n = 3, mean ± s.e.m.; Student’s t test; *P < 0.05, **P < 0.01). D and F, Representative HE-stained lung sections from the indicated groups at day 21. E and G, Representative Masson’s trichrome-stained lung sections from the indicated groups at day 21. Scale bars, 1000 μm (whole tissue) and 20 μm (enlarged areas).

To determine whether the antifibrotic effects of siArg1 or *Irf5* mRNA delivery were sustained beyond the early fibrotic phase, LNP-Mannose-encapsulated siArg1 or *Irf5* mRNA was administered intratracheally on day 7 after BLM challenge, and lungs were analyzed on day 21 (Figure 5A). At this later time point, BAL macrophages from both treatment groups continued to exhibit reduced CD206 expression compared with controls, indicating sustained suppression of the M2-like macrophage phenotype (Figure 5B). Consistently, the absolute number of M2 macrophages in BAL remained significantly reduced in both the siArg1 and *Irf5* mRNA groups compared with the control group (Figure 5C). Histopathological analysis further showed that both treatments attenuated lung fibrosis at day 21, as evidenced by reduced interstitial thickening and inflammatory cell infiltration on HE staining, together with decreased collagen deposition on Masson’s trichrome staining (Figure 5D-G).

## Discussion

**Figure 6:**
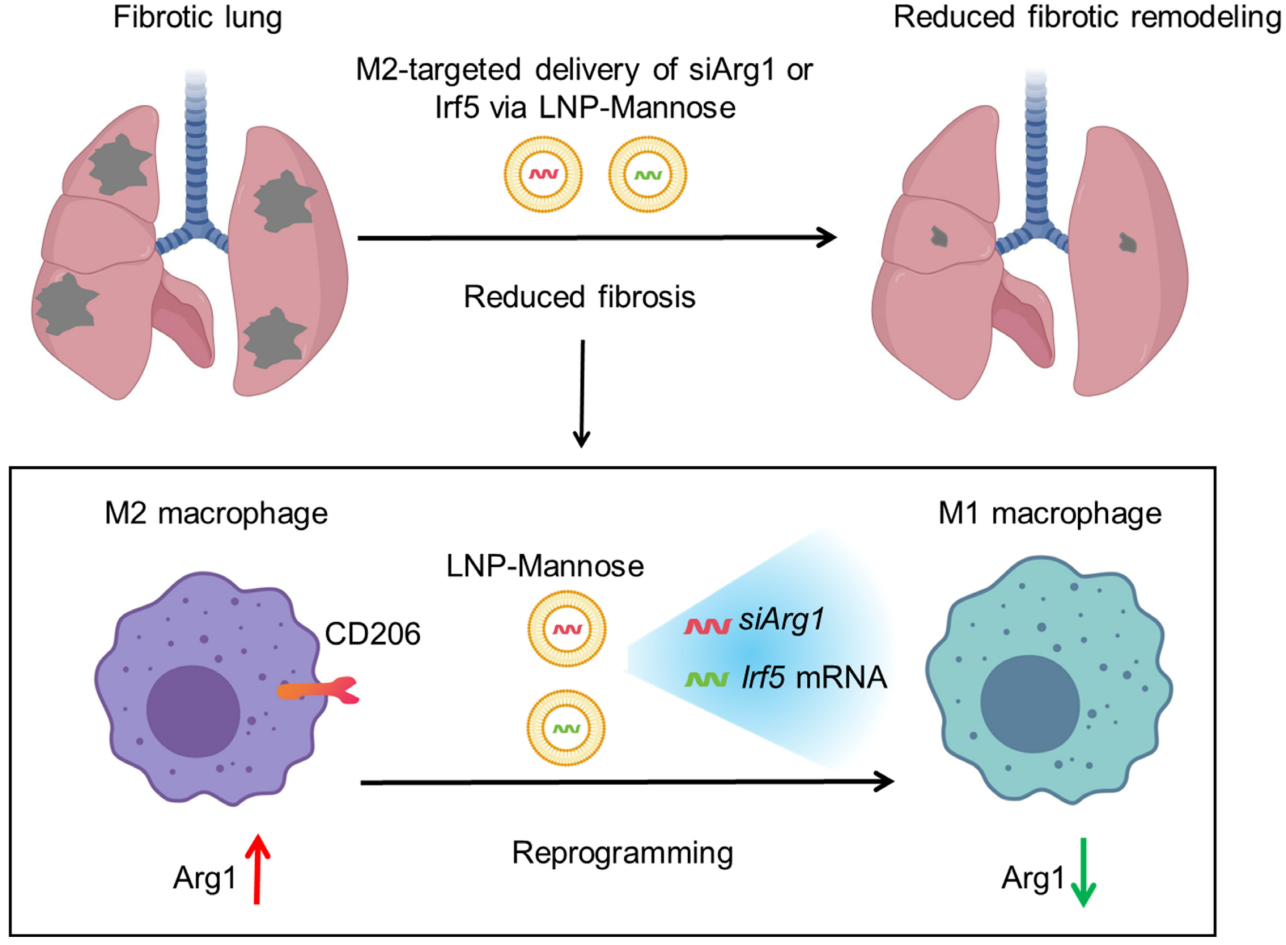
** Targeted RNA reprogramming of profibrotic macrophages in pulmonary fibrosis.** During pulmonary fibrosis, CD206⁺ M2 macrophages exhibit elevated Arg1 expression and enhanced arginine metabolism, supporting the generation of ornithine, proline, and hydroxyproline to fuel extracellular matrix production and fibrotic remodeling. LNP-Mannose preferentially target CD206⁺ macrophages in the lung to enable state-specific RNA delivery. Silencing Arg1 via siRNA destabilizes the profibrotic metabolic program and weakens M2 state stability, whereas targeted delivery of *Irf5* mRNA transcriptionally reprograms macrophages toward an inflammatory, antifibrotic functional state. Together, these state-directed RNA interventions illustrate how pathogenic macrophage programs can be dismantled and redirected to attenuate lung fibrosis.

Fibrosis represents a conserved pathological outcome of chronic injury across tissues, arising from persistent immune–stromal interactions that lock repair programs into a self-sustaining, matrix-producing state 2, 3, 22. Across organs, macrophages function as central integrators of environmental signals and key arbiters of fibrotic remodeling 6, 23, yet therapeutic strategies have struggled to selectively disrupt profibrotic macrophage programs without compromising essential immune functions. Here, we advance a distinct conceptual framework—state-level immune reprogramming—in which pathogenic macrophage states are therapeutically targeted, destabilized, and redirected rather than eliminated. Using pulmonary fibrosis as a model, we show that profibrotic macrophage identity is maintained by coupled metabolic and transcriptional programs, and that selective perturbation of these internal regulatory layers is sufficient to attenuate fibrotic persistence (Figure 6).

In idiopathic pulmonary fibrosis, arginine-related metabolic pathways are consistently upregulated and closely linked to collagen production and fibrotic remodeling 12, 24, 25. Enhanced arginine flux supports the generation of ornithine, proline, and hydroxyproline, thereby providing essential substrates for extracellular matrix synthesis 12, 24, 25. However, arginine metabolism is a fundamental and broadly required metabolic process, supporting vascular function, immune responses, and redox homeostasis across multiple cell types 26, 27. As a result, indiscriminate modulation of arginine metabolism carries a high risk of systemic disruption. These considerations highlight the need for precise, cell state–specific control of arginine-dependent metabolic programs, rather than global inhibition, in the development of effective antifibrotic therapies.

Within this metabolic landscape, our data identify Arg1 as a stage-specific regulator selectively enriched in macrophages during the transition from inflammation to fibrosis. Although Arg1 has been widely used as a marker of alternatively activated macrophages and linked to fibrotic pathology across tissues 12, 28, our integrated single-cell transcriptomic and metabolomic analyses support a more active role in stabilizing profibrotic macrophage identity. By coupling arginine utilization to downstream collagen-producing pathways, Arg1 functions as a metabolic constraint that reinforces a disease-supportive macrophage state.

This interpretation is supported by the observation that siArg1 treatment reduced ornithine and proline levels in lung tissue, accompanied by decreased TGF-β1, consistent with attenuation of profibrotic metabolic flux. In the siArg1 setting, the selective increase in IL-6, in the absence of clear changes in TNF-α, IL-1β, or IL-10, suggests that Arg1 silencing does not induce broad inflammatory activation, but instead promotes a more restricted remodeling of the local immune environment. Together, these findings strengthen the link between Arg1-associated metabolic remodeling and fibrosis attenuation *in vivo*, although they do not by themselves establish a fully cell-autonomous causal pathway within macrophages.

Disruption of Arg1 therefore destabilizes the profibrotic macrophage program, supporting a model in which these states are actively maintained through metabolic reinforcement rather than passively acquired. These findings position macrophage metabolism as a central driver of fibrotic persistence rather than a downstream consequence of tissue remodeling. Importantly, this state-targeted metabolic strategy may be extendable to other metabolic enzymes that stabilize pathogenic immune states, enabling selective reprogramming without globally perturbing essential metabolic processes.

Notably, the effect of Arg1 silencing on CD86 differed between the *in vitro* and *in vivo* settings. *In vitro*, Arg1 knockdown reduced CD86 expression under simplified culture conditions, whereas *in vivo* it was associated with increased CD86 expression in macrophages within the fibrotic lung. We interpret this difference as context-dependent, likely reflecting the influence of the complex injury-, inflammatory-, and stroma-associated signals present in the *in vivo* fibrotic microenvironment. Importantly, our interpretation of siArg1-mediated macrophage reprogramming does not rely solely on CD86, but is supported by the overall phenotypic, metabolic, and histological changes observed following treatment.

A key conceptual advance of this work is the separation of metabolic stabilization from transcriptional instruction in macrophage fate control. Thus, rather than representing two independent interventions, Arg1 silencing and *Irf5* mRNA delivery were used here as complementary strategies targeting distinct regulatory layers of macrophage state control: metabolic dependency and transcriptional instruction. Metabolic perturbation through Arg1 knockdown weakens the stability of the profibrotic state but does not impose a defined alternative identity. In contrast, enforced expression of the transcription factor IRF5 acts as a dominant instructive signal that actively drives macrophages toward an M1 functional program, consistent with its established role in inflammatory macrophage specification 14, 15.

Importantly, *Irf5* mRNA delivery was not only associated with marker-level changes, but also with coordinated alterations in the local cytokine and metabolic environment, including increased IL-6 and TNF-α, reduced TGF-β1, and decreased ornithine/proline levels. The increase in IL-6 and TNF-α likely reflects a shift away from a profibrotic macrophage program toward a more inflammatory activation state, consistent with IRF5's known role in macrophage polarization. Notably, this change occurred alongside reduced TGF-β1, lower ornithine/proline levels, and attenuated fibrosis, suggesting functional reprogramming rather than nonspecific inflammatory exacerbation. These findings support the view that transcriptional reprogramming by IRF5 extends beyond surface phenotype and is accompanied by broader functional remodeling within the fibrotic lung. More broadly, these results support a hierarchical architecture of macrophage plasticity in which transcription factors determine fate direction, while metabolic programs maintain state stability.

Macrophage reprogramming via nanoparticle-enabled delivery of IRF5 and IKKβ has previously been shown to reprogram tumor-associated macrophages and promote tumor regression in late-stage ovarian cancer models 16. Distinct from these cancer-focused studies, which primarily leverage inflammatory activation to enhance antitumor immunity, our work extends targeted IRF5-mediated macrophage reprogramming to a non-malignant, chronic fibrotic context. We show that targeted *Irf5* mRNA delivery alters pulmonary macrophage function and is associated with attenuation of lung fibrosis, indicating that the lung is a tractable site for *in vivo* macrophage reprogramming. Importantly, rather than broadly amplifying inflammation, this approach selectively rewires disease-supportive macrophage states that sustain fibrotic persistence.

We note several limitations of the current study. First, profibrotic macrophages were defined primarily based on CD206, together with Arg1, CD86, and iNOS as supporting markers. Although CD206 is widely used in fibrosis models, macrophage states *in vivo* are heterogeneous and continuous rather than strictly binary. Thus, the term “M2-like” is used here as an operational definition rather than a complete description of macrophage identity. Second, the present study did not include a direct comparison with current standard-of-care antifibrotic drugs such as pirfenidone or nintedanib. Such comparisons will be important in future preclinical studies to better evaluate the therapeutic potential and translational relevance of this strategy.

## Conclusion

Together, these findings support a state-level model of macrophage reprogramming in which pathogenic immune states are maintained by coupled metabolic and transcriptional circuits. In this context, the significance of our study lies not in re-establishing Arg1 or IRF5 as macrophage regulators, but in showing that these distinct regulatory axes can be therapeutically exploited through targeted RNA delivery to selectively remodel profibrotic macrophage states in the lung. Therapeutic intervention, therefore, need not depend on wholesale cell elimination or overly simplified binary polarization, but can instead focus on selectively perturbing the internal regulatory architecture that stabilizes disease-promoting states. This view aligns with emerging concepts of immune cell identity as a dynamic and modular property shaped by tissue context and regulatory state rather than fixed lineage boundaries. In summary, our work establishes targeted macrophage immune reprogramming as a conceptual framework for understanding and therapeutically targeting macrophage-driven fibrosis and suggests that coordinated metabolic destabilization and transcriptional instruction may offer a tractable strategy for intervening in chronic fibrotic disease.

## Methods

### Mice

All mouse experiments were approved by the Institutional Animal Care and Use Committee (IACUC) of the Guangzhou National Laboratory (approval numbers: GZLAB-AUCP-2025-10-A01). All mice were kept under specific pathogen-free conditions in the central animal facility of the Guangzhou National Laboratory. Male mice between 10 and 14 weeks of age were used in the experiments.

### Cell lines

HEK293T cells and RAW264.7 cells were maintained in Dulbecco’s modified Eagle’s medium (DMEM) supplemented with 10% fetal bovine serum (FBS) and 1% penicillin–streptomycin, and cultured at 37 °C in a humidified incubator with 5% CO₂.

### *In vitro* macrophage culture and polarization

Bone marrow–derived macrophages (BMDMs) were generated by culturing bone marrow cells in DMEM supplemented with 10% FBS and M-CSF (20 ng/mL) for 6 days. For M1 polarization, BMDMs were stimulated with LPS (20 ng/mL) and IFN-γ (20 ng/mL) for 24 h. For M2 polarization, BMDMs were stimulated with IL-4 (20 ng/mL) and IL-13 (20 ng/mL) for 24 h.

### LNP-Mannose formulation and characterization

Mannose-modified lipid nanoparticles (LNP-Mannose) were formulated based on a previously established LNP platform 21. To enable preferential targeting of M2 (CD206⁺) macrophages, DSPE–PEG2000–mannose was incorporated. The lipid components—DLin-MC3-DMA, cholesterol, DOPE, and DSPE–PEG2000–Mannose—were combined at a molar ratio of 35:46.5:16:2.5.

Z-average diameter, polydispersity index (PDI), and zeta potential of nanoparticles were analyzed using a Zetasizer Ultra (Malvern Instruments, U.K.).

### mRNA synthesis and purification

Modified linear mCherry and mouse *Irf5* mRNAs were designed to include a protein-coding sequence flanked by 5′ and 3′ untranslated regions (UTRs) derived from human β-globin, a Cap 1 structure, and a 120-nucleotide poly(A) tail. mRNAs were synthesized *in vitro* by T7 RNA polymerase–mediated transcription from linearized DNA templates, with complete substitution of uridine and cytidine residues by pseudouridine and 5-methylcytidine, respectively, throughout the transcript. Following transcription, DNA templates were removed by DNase I treatment for 15 min. The resulting linear mRNAs were subsequently purified using the Monarch® RNA Cleanup Kit according to the manufacturer’s instructions.

### BLM-induced pulmonary fibrosis model

Pulmonary fibrosis was induced by intratracheal instillation of bleomycin (BLM; 2.5 U/kg in 50 μL PBS; Selleck Chemicals) under anesthesia. Control mice received an equal volume of PBS. Lung tissues were harvested at the indicated time points after BLM administration for subsequent analyses.

### Single-cell RNA sequencing analysis

Lung tissues were harvested at days 0 (naive control), 3, 10, and 21 after bleomycin challenge and processed to generate single-cell suspensions by enzymatic digestion and mechanical dissociation. Single-cell libraries were prepared using the Chromium Single Cell 3′ v3 platform (10x Genomics) according to the manufacturer’s instructions. Libraries were sequenced on an Illumina NovaSeq 6000 platform with 150-bp paired-end reads. Raw count matrices were processed using Seurat. Low-quality cells were excluded based on gene number and mitochondrial gene content. Data were normalized, scaled, and subjected to dimensionality reduction and clustering. Cell types were annotated based on canonical marker gene expression. Arg1 expression across cell types and time points was visualized using violin plots. All single-cell analyses were performed using gene expression matrices derived from in-house scRNA-seq datasets.

### Detection and analysis of targeted metabolites

Lung tissues were collected from control and BLM-treated mice at day 21 after injury. Samples were thawed, homogenized, and 50 mg of each sample was extracted with 500 μL of 70% methanol/water, vortexed (3 min), and centrifuged (12,000 rpm, 10 min, 4 °C). Supernatants were chilled at -20 °C for 30 min, re-centrifuged, and clarified through a protein precipitation plate prior to LC–MS/MS analysis. Metabolite profiling was performed on an ExionLC AD UPLC system coupled to a QTRAP 6500+ mass spectrometer (Sciex) using either an ACQUITY UPLC HSS T3 C18 column or a BEH Amide column with optimized gradient elution. Mass spectrometry was conducted in positive and negative ESI modes using scheduled multiple reaction monitoring (MRM). Data acquisition was performed with Analyst 1.6.3, and metabolite quantification was carried out using MultiQuant 3.0.3 with optimized declustering potentials and collision energies for each metabolite. These metabolites were detected by MetWare (http://www.metware.cn/, Wuhan MetWare Biotechnology Co., Ltd.) based on the AB Sciex QTRAP 6500 LC-MS/MS platform.

Differentially regulated metabolites were identified based on variable importance in projection (VIP) scores from orthogonal partial least squares–discriminant analysis (OPLS-DA) and absolute log₂ fold change. OPLS-DA was performed using the MetaboAnalystR package in Rafter log₂ transformation and mean centering of the data. Model robustness was assessed by 200-permutation testing to minimize overfitting. Identified metabolites were annotated against the KEGG compound database and mapped to KEGG pathways. Metabolite set enrichment analysis (MSEA) was conducted using a hypergeometric test to determine significantly enriched pathways. Absolute quantification of targeted metabolites is provided in Supplementary Table 1.

### *In vitro* LNP-Mannose transfection

For cytotoxicity assays, RAW264.7 cells (1 × 10⁵ cells per well) were transfected with LNP-Mannose encapsulating up to 5 μg mCherry mRNA. Apoptosis was assessed 24 h later by flow cytometry using Annexin V and propidium iodide staining.

For macrophage reprogramming experiments, M2-polarized BMDMs (1 × 10⁵ cells per well) were transfected with LNP-Mannose–encapsulated siArg1 (50 nM), siCtrl (50 nM), *Irf5* mRNA (5 μg), or vehicle control. Cells were analyzed 48 h after transfection.

### *In vivo* RNA delivery

Mice received 50 μL of LNP-Mannose–encapsulated siArg1 (1 nmol), Irf5 mRNA (20 μg), or corresponding control formulations by intratracheal administration at day 7 after BLM-induced lung injury. Lungs were harvested for analysis at day 14 or day 21 after BLM administration, as indicated.

### Quantitative real-time PCR

RNA extraction was performed with the FastPure Cell/Tissue Total RNA Isolation Kit V2 (Cat# RC112-01, Vazyme, China). cDNA was synthesized using the HiScript III RT SuperMix for qPCR (+gDNA wiper) kit (Cat# R323-01, Vazyme, China). Quantitative PCR (qPCR) was carried out with SYBR® Green Real-Time PCR Master Mix (Cat# QPK-201, Toyobo, Japan) on a QuantStudio™ 3 Real-Time PCR system (Applied Biosystems, USA). Primer sequences (5′–3′) used in this study are listed below: Arg1-F: CATTGGCTTGCGAGACGTAGAC; Arg1-R: GCTGAAGGTCTCTTCCATCACC; Cd86-F: ACGTATTGGAAGGAGATTACAGCT; Cd86-R: TCTGTCAGCGTTACTATCCCGC; Nos2-F: GAGACAGGGAAGTCTGAAGCAC; Nos2-R: CCAGCAGTAGTTGCTCCTCTTC; Mrc1-F: GTTCACCTGGAGTGATGGTTCTC; Mrc1-R: AGGACATGCCAGGGTCACCTTT; 18S-F: CGAAAGCATTTGCCAAGAAT; 18S-R: AGTCGGCATCGTTTATGGTC. The expression of target genes was normalized using 18S rRNA as an internal control.

### Apoptosis assay

Apoptosis was analyzed using the BD Pharmingen™ FITC Annexin V Apoptosis Detection Kit (cat. 556547). Cells were stained with FITC–Annexin V and propidium iodide in Annexin V binding buffer and analyzed by flow cytometry. Early apoptotic cells were defined as Annexin V⁺PI⁻ and late apoptotic/necrotic cells as Annexin V⁺PI⁺.

### Western blotting

Protein samples were extracted using RIPA buffer containing protease inhibitors, and protein concentrations were measured by BCA assay. Equal amounts of protein were subjected to SDS–PAGE and transferred to nitrocellulose membranes. Membranes were blocked with 5% milk and incubated with primary antibodies (Anti-Arginase 1 Mouse Monoclonal Antibody (Cat# sc-271430, Santa Cruz); Anti-IRF5 Rabbit Antibody (Cat# A1149, ABclonal); Anti-GAPDH Mouse Monoclonal Antibody (Cat# TA802519, ORIGENE)) overnight at 4 °C, followed by incubation with HRP-conjugated secondary antibodies (Cat# 7074S and Cat# 7076S, Cell Signaling Technology) for 1 h at room temperature. Signals were detected using ECL reagents and quantified using ImageJ.

### Flow cytometry

Single-cell suspensions of lung tissue were prepared as previously described 29. Cells were incubated with purified rat anti-mouse CD16/CD32 antibody (Mouse BD Fc Block, clone 2.4G2; BD Pharmingen) to block Fc receptors, followed by surface staining with the following antibodies: BV421 rat anti-mouse CD45 (clone 30-F11; BD Horizon), BV510 rat anti-mouse Ly6G (clone 1A8; BD Horizon), Alexa Fluor 488 anti-mouse CD206 (clone C068C2; BioLegend), Brilliant Violet 605 anti-mouse CD86 (clone PO3; BioLegend), Alexa Fluor 700 anti-mouse MerTK (clone DS5MMER; Invitrogen), Brilliant Violet 711 anti-mouse CD64 (clone X54-5/7.1; BioLegend), and PE-Vio770 anti-mouse F4/80 (clone REA126; Miltenyi Biotec).

Macrophages were defined as CD45⁺CD64⁺MerTK⁺ cells, or alternatively as CD45⁺F4/80⁺ cells in some experiments. CD206⁺ macrophages were classified as M2-like, whereas CD206⁻ macrophages were classified as M1-like. PMNs were defined as CD45⁺F4/80⁻Ly6G⁺ cells.

For intracellular staining, cells were fixed and permeabilized after surface marker staining as previously described 30. Rabbit anti-iNOS polyclonal antibody (Cat# ab3523; Abcam) and mouse anti-Arginase-1 monoclonal antibody (Cat# sc-271430; Santa Cruz Biotechnology) were used as primary antibodies. Donkey anti-mouse Alexa Fluor 555 (Cat# ab150106; Abcam) and donkey anti-rabbit Alexa Fluor 647 (Cat# ab150155; Abcam) were used as secondary antibodies.

Flow cytometric data were acquired using a CytoFLEX S flow cytometer (Beckman Coulter) or a BD LSRFortessa X-20 cell analyzer (BD Biosciences) and analyzed with FlowJo software (Tree Star). In some experiments, pulmonary PMNs were sorted using a BD FACSAria III cell sorter (BD Biosciences).

### ELISA

Metabolite and cytokine levels in lung homogenates were quantified using commercial ELISA kits according to the manufacturers’ instructions. Briefly, lung tissues were homogenized on ice in PBS and centrifuged to remove debris. The clarified supernatants were collected for subsequent analysis. Concentrations were determined based on standard curves generated for each analyte and, where indicated, normalized to total protein content in the tissue lysates. Mouse ELISA kits for arginine, ornithine, and proline were purchased from Beijing Huabodeyi Biology Science and Technology Co., Ltd. Mouse ELISA kits for TGF-β1, PDGF-BB, TNF-α, IL-6, IL-1β, and IL-10 were purchased from MultiSciences (Lianke) Biotech Co., Ltd.

### Histology

In some experiments, the left lung, liver, heart, spleen, and kidney were fixed by inflation in 10% formalin, dehydrated in ethanol, embedded in paraffin, and cut into 5 μm sections for histological analysis. Tissue sections were stained with HE stain kit (Cat# G1005, Servicebio, China) or Masson’s trichrome stain kit (Cat# G1006, Servicebio). The images were captured with Evident Slidescanner Slideview VS200 (Olympus, Japan). Images were initially analyzed using OlyVIA 4.1 software (Olympus, Japan) and further processed and analyzed with ImageJ (National Institutes of Health, USA).

### Immunofluorescence staining

Formalin-fixed, paraffin-embedded lung tissue sections underwent antigen retrieval with Tris-EDTA buffer (pH 9.0, 10 mM Tris base, 1 mM EDTA). Sections were blocked with normal donkey serum (Cat# SL050, Solarbio, China) for 1 h at room temperature, incubated with primary antibodies (Anti-iNOS Mouse Monoclonal Antibody (Cat# 610329, BD); Anti-CD68 Rabbit Polyclonal Antibody (ab125212, Abcam); Anti-Arginase 1 Mouse Monoclonal Antibody (Cat# sc-271430, Santa Cruz); Anti-CD31 Rat Monoclonal Antibody (Cat# ab56299, Abcam); Anti-EpCAM Rabbit Polyclonal Antibody (Cat# ab71916, Abcam); Anti-α-SMA Mouse Monoclonal Antibody (Cat# A2547, Sigma); Anti-PDGFR-β Rabbit Polyclonal Antibody (Cat# SC339, Santa Cruz) ) at 4 ^o^C overnight, followed by incubation with 1/500 dilution of secondary antibodies (Donkey Anti-Rabbit-Alexa Fluor 488 (Cat# ab150073, Abcam), Donkey Anti-Mouse-Alexa Fluor 488 (Cat# ab150105, Abcam), Donkey Anti-Rabbit-Alexa Fluor 555 (Cat# ab150074, Abcam), Donkey Anti-Mouse-Alexa Fluor 647 (Cat# ab150107, Abcam) ) for 1 h at room temperature. 1 μg/mL DAPI (Cat# 10236276001, Roche) was used for nuclear counterstaining. ProLong™ Diamond Antifade Mountant with DAPI (Cat# P36930, Invitrogen) was used as mounting medium. Images were captured with Evident Slidescanner Slideview VS200 (Olympus, Japan). Images were further processed and analyzed using ImageJ (NIH).

### Statistics

For statistical analyses, we used Prism 10.0 (GraphPad Software, Inc., San Diego, CA). Statistical significance was assessed using Student’s t test or one-way ANOVA as indicated. A P value <0.05 was considered statistically significant.

## Supplementary Material

Supplementary figures.

Supplementary table 1.

## Data Availability

Targeted metabolomics data are provided in Supplementary Table 1.

## Abbreviations

Arg1: arginase-1
BAL: bronchoalveolar lavage
BLM: bleomycin
BMDMs: bone marrow-derived macrophages
CD206: cluster of differentiation 206
CD68: cluster of differentiation 68
ECM: extracellular matrix
IL-1β: interleukin-1 beta
IL-6: interleukin-6
IL-10: interleukin-10
IRF5: interferon regulatory factor 5
IT: intratracheal
LNP: lipid nanoparticle
LNP-Mannose: mannose-functionalized lipid nanoparticle
M1: classically activated macrophage
M2: alternatively activated macrophage
PDI: polydispersity index
PDGF-BB: platelet-derived growth factor-BB
TGF-β1: transforming growth factor-beta 1
TNF-α: tumor necrosis factor-alpha
