# Supplementary material for: Targeted RNA Therapy Reprograms Fibrotic Macrophages to Reverse Pulmonary Fibrosis
Source: Theranostics. 2026 Jun 4;16(13):7308–23. doi: 10.7150/thno.132296 (PMC13294990; doi:10.7150/thno.132296)
Supplement: Supplementary file 1 — Supplementary figures. [file thnov16p7308s1.pdf]

Supplemental materials

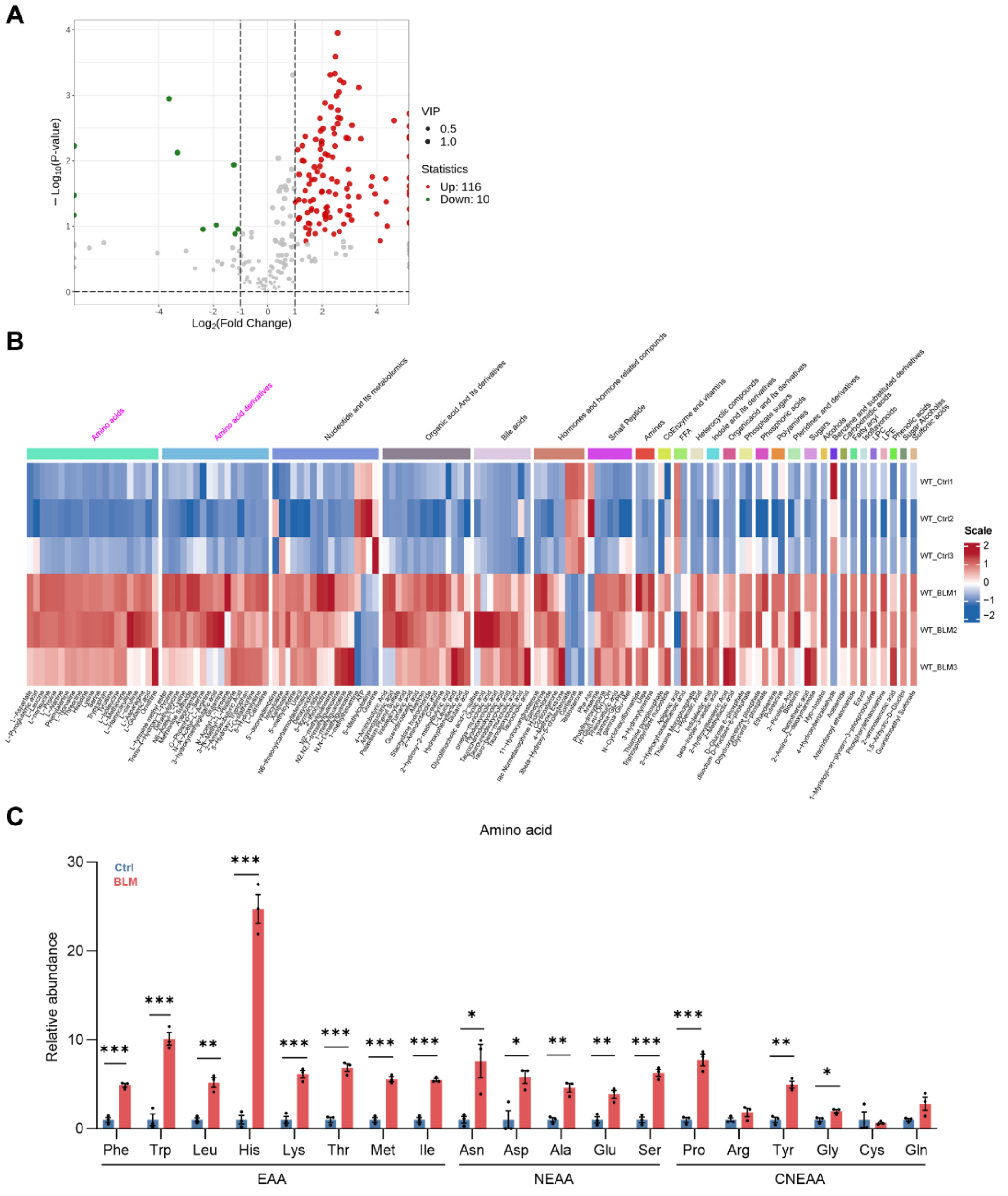

Figure S1. Targeted metabolomic profiling reveals elevated amino acid metabolism in fibrotic lungs.

A, Volcano plot showing differentially abundant metabolites between control and BLM-treated mice at day 21. B, Heatmap depicting the relative abundance of metabolites in control and BLM-treated mice at day 21. C, Bar plots showing the relative abundance of amino acids in lung tissues from mice treated with bleomycin or vehicle control at day 21 post-treatment. Data are presented as mean ± s.e.m. (n = 3). Statistical significance was determined by Student's t test; *P < 0.05, **P < 0.01, ***P < 0.001. EAA, essential amino acids; NEAA, non-essential amino acids; CNEAA, conditionally non-essential amino acids.

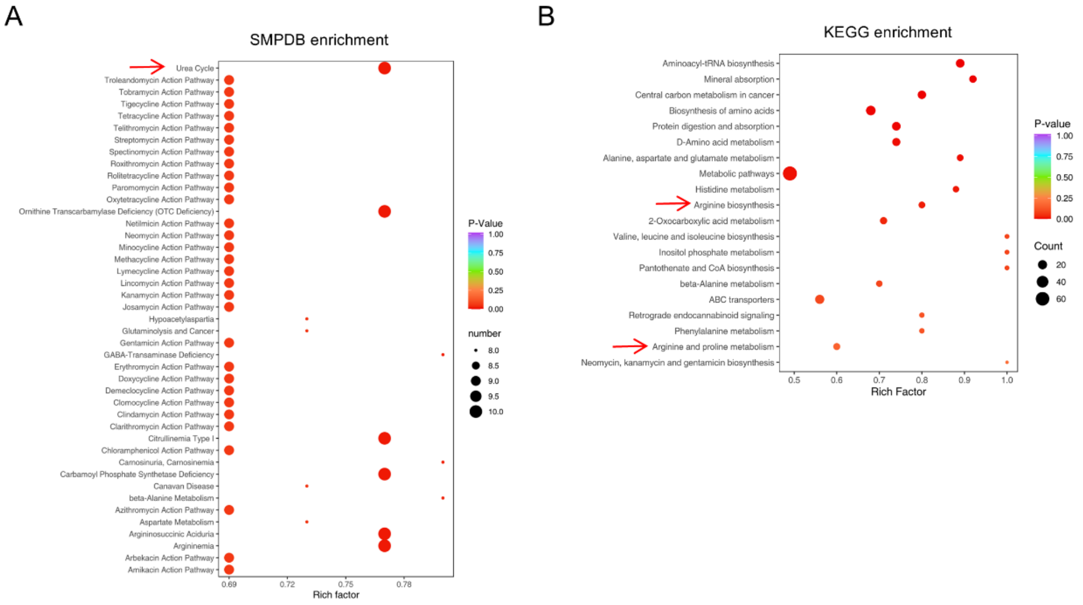

Figure S2. Pathway enrichment analyses highlight arginine-centered metabolic reprogramming in fibrotic lungs.

A, Small Molecule Pathway Database (SMPDB) primary enrichment analysis of differentially abundant metabolites, showing enrichment of arginine-associated metabolic pathways. B, KEGG pathway enrichment analysis of differentially abundant metabolites, highlighting arginine metabolism–related pathways.

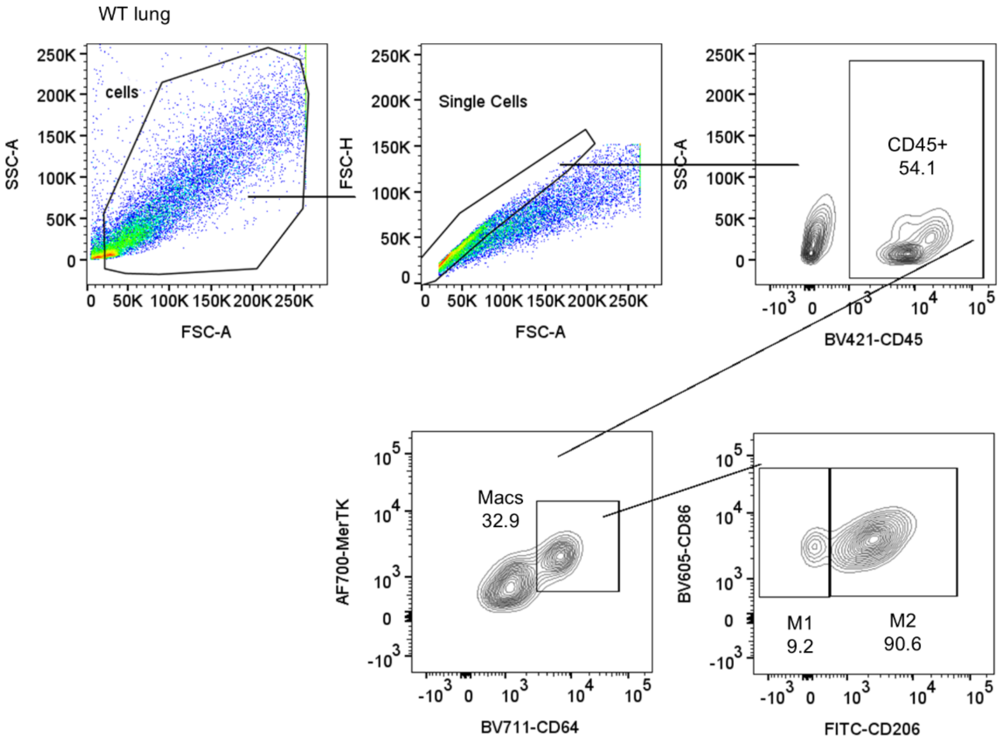

Figure S3. Flow cytometric gating strategy for M1 and M2 macrophage subsets in lung tissue.

Representative flow cytometry plots showing the sequential gating strategy used to identify lung macrophage subsets. Following exclusion of debris and doublets, $CD45^+$ leukocytes were gated, and macrophages were defined as $CD64^+MerTK^+$ cells. Macrophages were further subdivided into M1 ($CD206^-$) and M2 ($CD206^+$) populations based on CD206 expression.

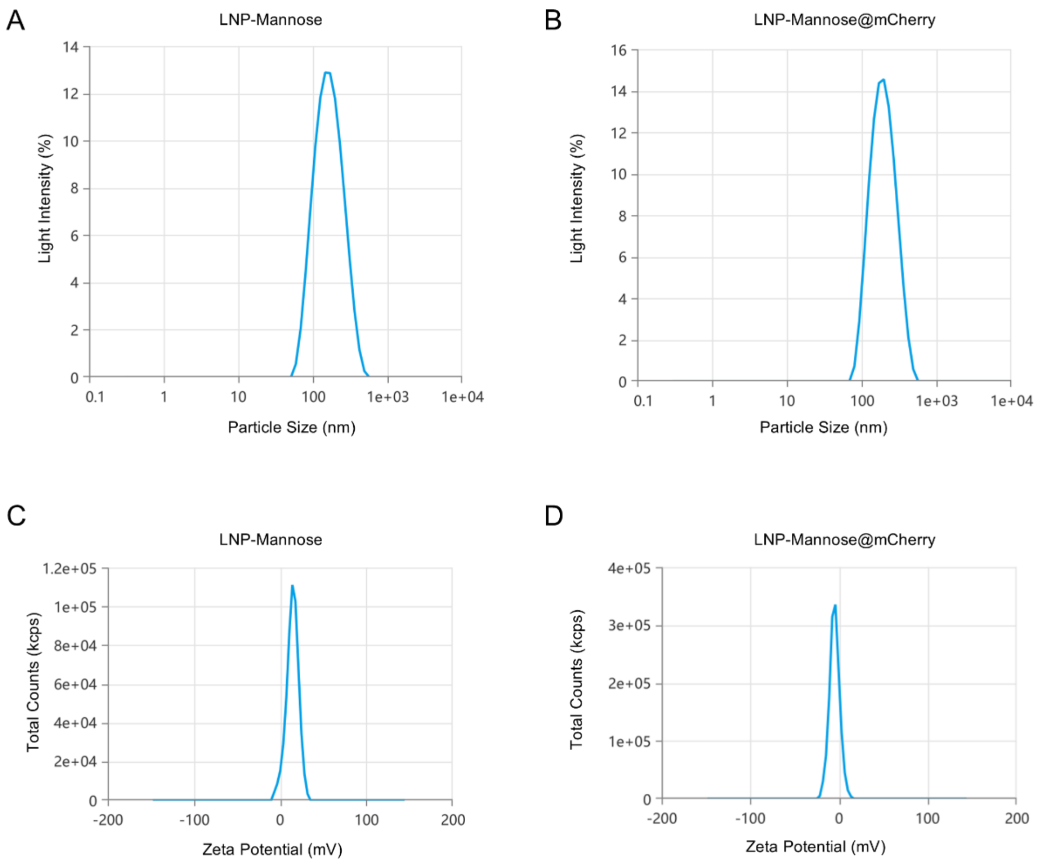

E

Group	LNP-Mannose	LNP-Mannose@mCherry
**Size (nm)**	147.1 ± 0.2	184.7 ± 1.6
**Polydispersity index (PDI)**	0.152 ± 0.011	0.195 ± 0.024
**Zeta potential (mV)**	14.04 ± 0.66	-4.76 ± 0.56

Figure S4. Physicochemical characterization of LNP-Mannose and LNP-Mannose@mCherry.

A, Representative intensity-weighted size distribution of LNP-Mannose. B, Representative intensity-weighted size distribution of LNP-Mannose@mCherry. C, Representative zeta potential distribution of LNP-Mannose. D, Representative zeta potential distribution of LNP-Mannose@mCherry. E, Summary table showing the Z-average size, polydispersity index (PDI), and zeta potential of LNP-Mannose and LNP-Mannose@mCherry. Data are presented as mean ± s.e.m. (n = 3).

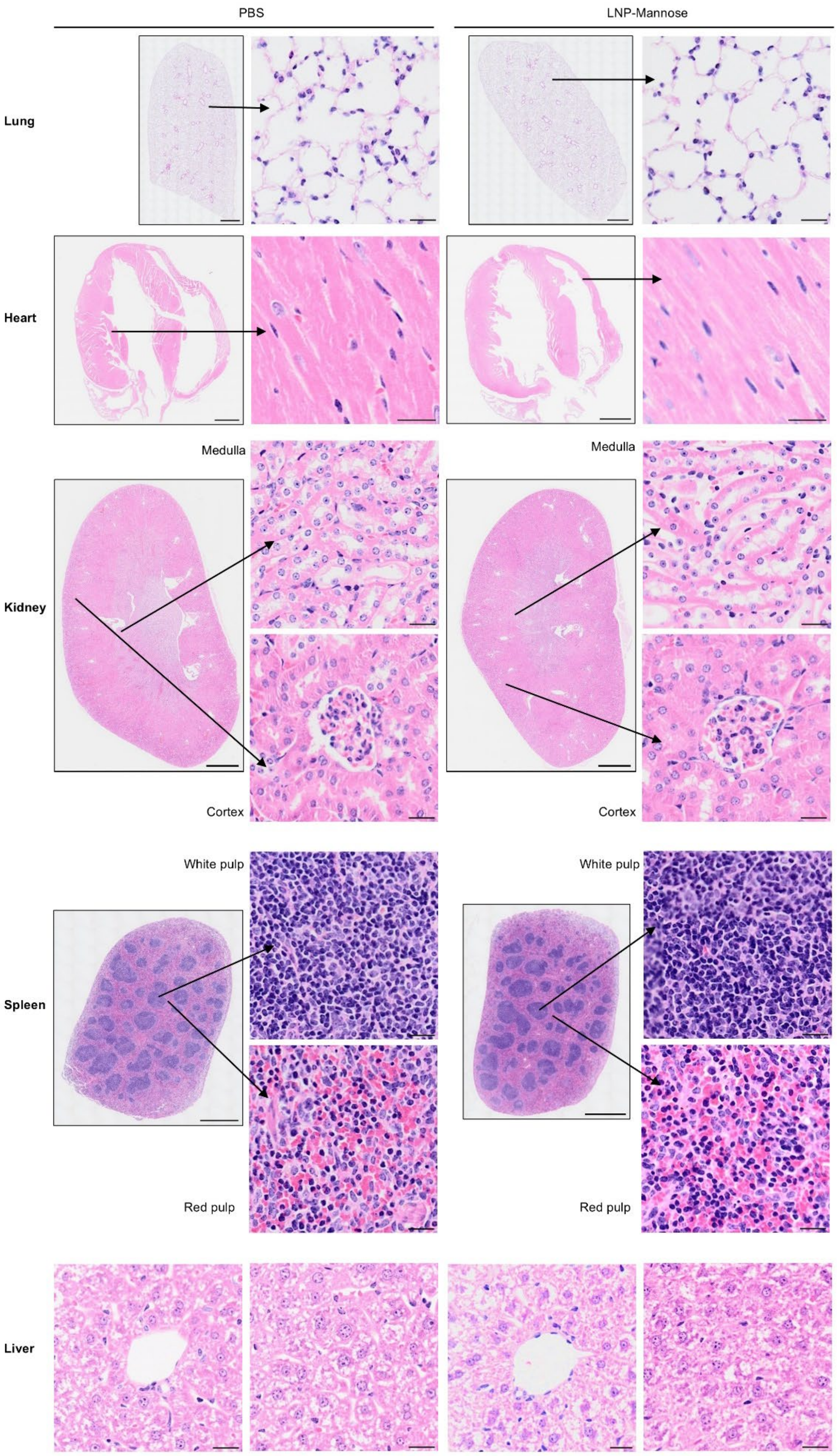

Figure S5. In vivo safety evaluation of LNP-Mannose after intratracheal administration.

Representative hematoxylin and eosin (H&E) staining of heart, liver, spleen, lung, and kidney sections collected 24 h after intratracheal administration of PBS or LNP-Mannose. No obvious histopathological differences were observed between the two groups. Scale bars, 1000 μm (whole tissue) and 20 μm (enlarged areas).

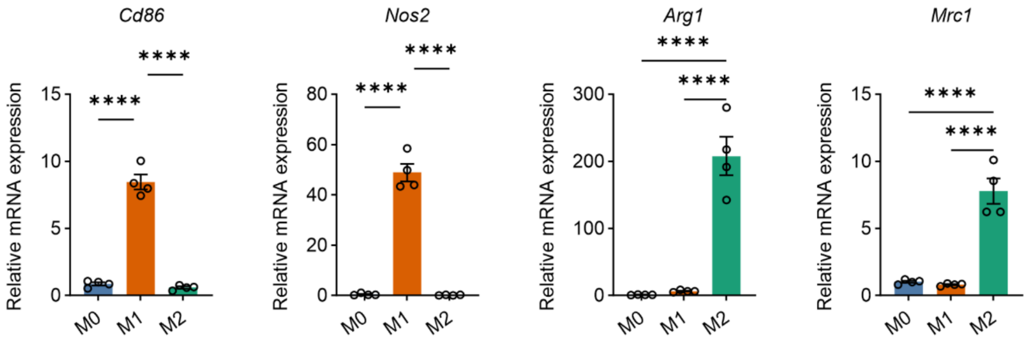

Figure S6. Expression of M1- and M2-associated genes in M0, M1, and M2 BMDMs.

Expression of Cd86, Nos2, Arg1, and Mrc1 in M0, M1, and M2 BMDMs. BMDMs were left unstimulated (M0) or polarized toward the M1 or M2 phenotype, and gene expression was measured by qPCR. Data are presented as mean ± s.e.m. Statistical significance was determined by One-Way ANOVA; ****P < 0.0001.

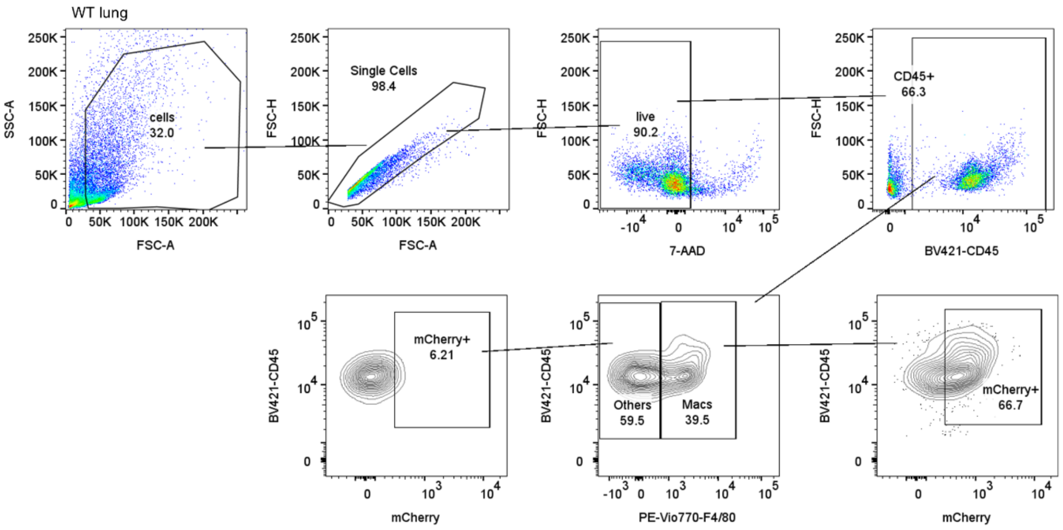

Figure S7. Flow cytometric gating strategy to assess macrophage-specific mRNA delivery by LNP-Mannose in lung tissue.

Representative flow cytometry plots illustrating the sequential gating strategy used to evaluate cell type–specific mRNA delivery following LNP-Mannose administration. After exclusion of debris and doublets, live CD45$^+$ leukocytes were gated, followed by identification of macrophages based on F4/80$^+$ expression. mRNA delivery was assessed by detection of mCherry fluorescence within the macrophage gate and compared with other immune cell populations as indicated.

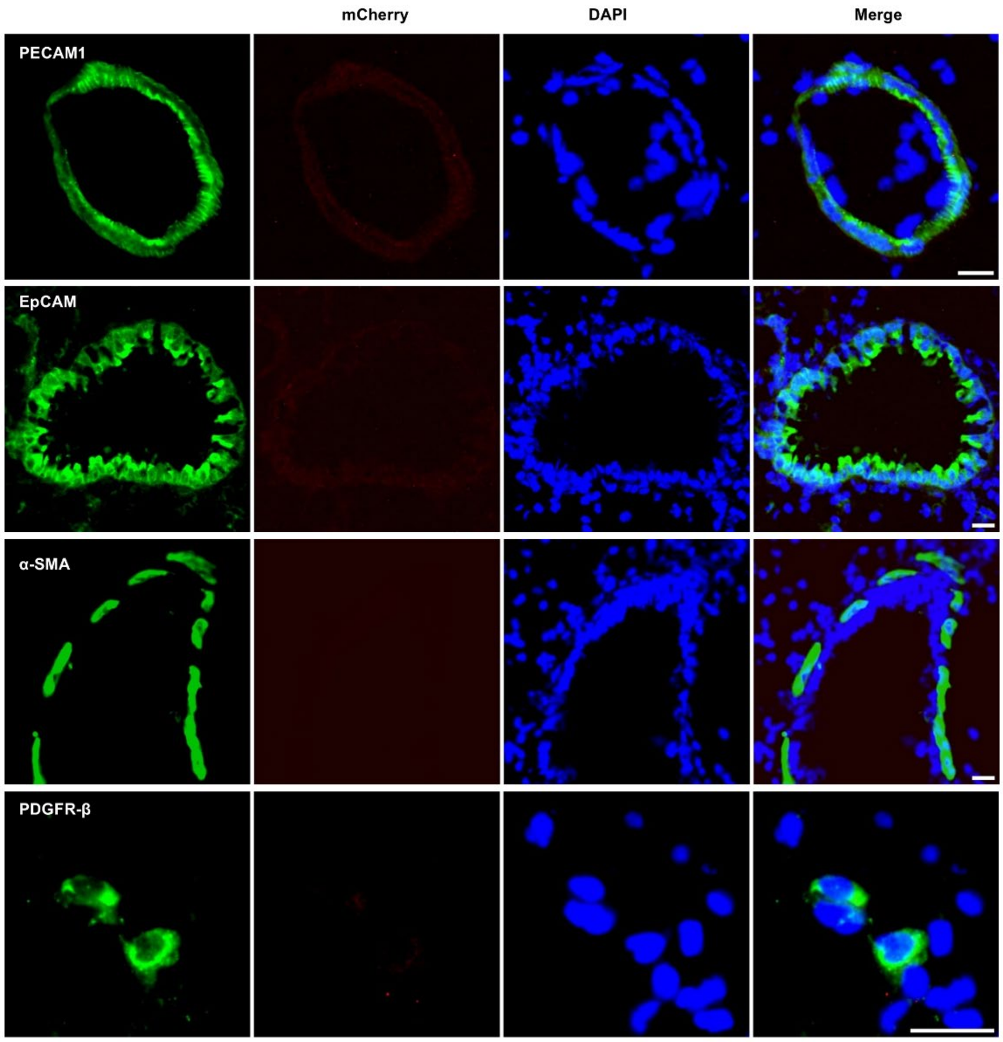

Figure S8. mCherry distribution in non-macrophage lung cell populations after LNP-Mannose delivery.

Representative immunofluorescence staining of lung sections after LNP-Mannose delivery showing mCherry signal in combination with PECAM1, EpCAM, α-SMA, or PDGFR-β. Scale bars, 20 μm.

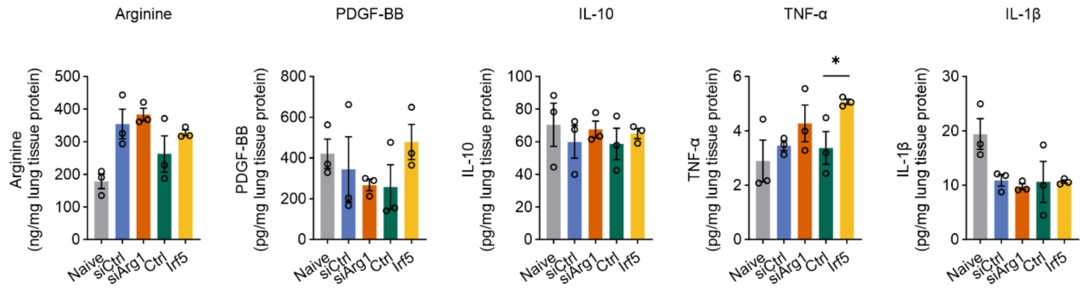

Figure S9. Arginine, PDGF-BB, IL-10, TNF-α, and IL-1β levels in lung tissue homogenates after siArg1 or *Irf5* mRNA treatment.

Quantification of arginine, PDGF-BB, IL-10, TNF-α, and IL-1β levels in lung tissue homogenates from naive mice and bleomycin-treated mice receiving LNP-Mannose alone (control for *Irf5* mRNA treatment), or LNP-Mannose–encapsulated siCtrl, siArg1, or *Irf5* mRNA on day 7 after bleomycin administration, with analysis performed on day 14. Levels were normalized to total protein concentration in lung homogenates. Data are shown as mean ± s.e.m. ($n = 3$). Student's t test; $*P < 0.05$.
